# A Narrative Review on the Promising Potential of Graphene in Vaccine Design: Evaluating the Benefits and Drawbacks of Carbon Nanoplates in Nanovaccine Production

**DOI:** 10.3390/vaccines12060660

**Published:** 2024-06-14

**Authors:** Hadi Zare-Zardini, Elham Saberian, Andrej Jenča, Andrej Jenča, Adriána Petrášová, Janka Jenčová

**Affiliations:** 1 Department of Biomedical Engineering, Meybod University, Meybod 89616-99557, Iran; 2 Klinika and Akadémia Košice Bacikova, Pavol Jozef Šafárik University, 040 01 Kosice, Slovakia; 3 Klinika of Stomatology and Maxillofacial Surgery Akadémia Košice Bacikova, UPJS LF, 040 11 Kosice, Slovakiaadriana.petrasova@upjs.sk (A.P.);

**Keywords:** graphene, vaccine, adjuvant, immune response

## Abstract

Graphene, a two-dimensional material consisting of a single layer of carbon atoms arranged in a honeycomb lattice, has shown great potential in various fields, including biomedicine. When it comes to vaccine development, graphene can offer several advantages due to its unique properties. Potential applications of graphene in vaccine development include improved vaccine delivery, adjuvant properties, improved vaccine stability, improved immune response, and biosensing capabilities. Although graphene offers many potential benefits in vaccine development, there are also some drawbacks and challenges associated with its use. Although graphene shows promising potential for vaccine development, overcoming the challenges and limitations associated with its use is critical to realizing its full potential in the field of immunization. Further research and development efforts are needed to overcome these drawbacks and take advantage of graphene for improved vaccine formulations. In this review, we focus on the advantages and disadvantages of graphene for vaccine development.

## 1. Introduction

Vaccines are one of the most important and effective public health interventions, playing a crucial role in preventing and controlling infectious diseases [1].

While vaccines are highly effective public health interventions, there are some limitations and challenges associated with vaccine design and development:

- Pathogen variability: Many pathogens, such as influenza virus and HIV, have the ability to rapidly mutate and change their antigenic properties. This variability can make it challenging to develop broadly effective vaccines that can provide protection against multiple strains or variants of the same pathogen. Vaccine developers must continuously monitor pathogen evolution and update vaccine compositions to maintain effectiveness.
- Immune system complexity: The human immune system is highly complex, and individual responses to vaccines can vary significantly based on factors such as age, genetics, and underlying health conditions. Certain populations, like the elderly or immunocompromised individuals, may have a weaker or less robust immune response to vaccines. Designing vaccines that can elicit a consistent and protective immune response across diverse populations is an ongoing challenge.
- Vaccine safety and adverse events: Ensuring the safety of vaccines is of paramount importance, and vaccine developers must thoroughly evaluate the potential for adverse events or side effects. Even rare adverse events can raise public concerns and affect vaccine acceptance, necessitating robust safety monitoring and communication strategies.
- Vaccine stability and storage: Many vaccines require specific storage conditions, such as cold-chain maintenance, to preserve their potency and efficacy. Maintaining the cold chain can be particularly challenging in resource-limited settings or areas with limited infrastructure, which can impact vaccine availability and distribution. Developing more thermostable vaccine formulations or alternative delivery methods can help address these challenges.
- Regulatory and ethical considerations: Vaccine development and approval processes are subject to strict regulatory requirements to ensure safety and efficacy. Navigating the regulatory landscape and addressing ethical concerns, such as equitable access and fair distribution, can add complexity to the vaccine development process.
- Vaccine hesitancy and misinformation: Vaccine hesitancy, driven by factors such as misinformation, distrust, and personal beliefs, can hinder the uptake and effectiveness of vaccines. Addressing vaccine hesitancy requires a multifaceted approach, including effectively communicating, providing education, and addressing the underlying concerns of the public [2,3,4].

Despite these limitations, ongoing research and innovation in vaccine design, production, and delivery continue to address these challenges and contribute to the overall success of vaccination programs worldwide. The application of nanotechnology in vaccine design is an active area of research, with ongoing efforts to translate these innovative approaches into real-world vaccine products. Continued advancements in nanomaterials, formulation development, and clinical testing will be crucial for unlocking the full potential of nanotechnology in vaccine design and optimization [5,6,7]. In this review article, we evaluate the advantages and disadvantages of graphene materials in vaccine design.

## 2. Graphene Material Structure

**Figure 1:**
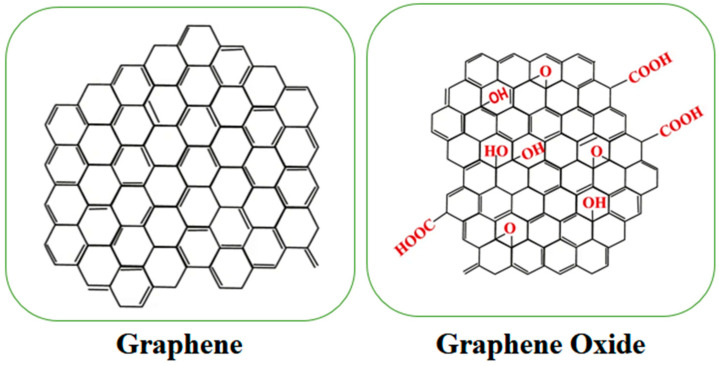
Structure of graphene and graphene oxide.

Graphene and graphene oxide are two-dimensional (2D) allotropes of carbon with a unique atomic structure and remarkable physical, chemical, and electronic properties (Figure 1). Graphene and graphene oxide are single layers of carbon atoms arranged in a hexagonal, honeycomb-like lattice [8,9]. Each carbon atom in graphene is covalently bonded to three neighboring carbon atoms, forming a planar, sp^2^-hybridized structure. The carbon–carbon bond length in graphene is approximately 0.142 nanometers (nm), resulting in a highly stable and rigid structure [10,11]. A high surface area, hydrophilicity, tunable electronic properties, and mechanical strength are the main properties of graphene oxide. There are several methods for the preparation of graphene oxide, including the Hummer method, the modified Hummer method, and green synthesis [12,13].

## 3. Graphene and Graphene Oxide Properties

### 3.1. Graphene

#### 3.1.1. Mechanical Properties

Graphene is one of the strongest materials known, with a tensile strength of around 130 gigapascals (GPa). It is also highly flexible and can withstand large deformations without breaking. Graphene’s high strength-to-weight ratio makes it an attractive material for various applications, such as reinforcing composites [14].

#### 3.1.2. Electrical Properties

Graphene is an excellent electrical conductor, with high electron mobility at room temperature (up to 200,000 cm^2^/Vs). It exhibits unique electronic properties, including the ability to support the flow of electrons as if they have no mass (massless Dirac fermions). Graphene’s high electrical conductivity and ability to control the flow of electrons make it promising for electronic and optoelectronic applications [15].

#### 3.1.3. Thermal Properties

Graphene has exceptional thermal conductivity, with values of up to 5000 watts per meter-Kelvin (W/m-K) at room temperature. This high thermal conductivity is attributed to the efficient transfer of phonons (lattice vibrations) through the planar structure of graphene [16].

#### 3.1.4. Optical Properties

Graphene is nearly transparent, absorbing only 2.3% of visible light due to its atomic thickness. The optical properties of graphene can be tuned by modifying its structure, doping, or applying external fields. These properties make graphene suitable for various optoelectronic applications, such as transparent electrodes and photodetectors [17].

#### 3.1.5. Surface Area and Adsorption

Graphene has a large specific surface area, with a theoretical value of up to 2630 square meters per gram (m^2^/g). This high surface area, combined with its unique physicochemical properties, allows graphene to effectively adsorb and interact with various molecules and materials [18].

#### 3.1.6. Graphene Applications

The exceptional properties of graphene have led to a wide range of potential applications in fields such as electronics, energy storage, sensors, composites, and biomedical engineering. Ongoing research continues to explore new ways to leverage the unique characteristics of graphene for innovative solutions. Some of the key applications of graphene include electronics and optoelectronics, energy storage and conversion, biomedical applications, composites and structural materials, water purification and environmental applications, thermal management, lubrication and tribology, and aerospace and defense [19,20,21].

### 3.2. Graphene Oxide

#### 3.2.1. High Surface Area

Graphene oxide has a high surface area due to its two-dimensional structure, which makes it an excellent material for applications such as energy storage, catalysis, and adsorption.

#### 3.2.2. Hydrophilicity

The presence of oxygen-containing functional groups on the surface of graphene oxide makes it hydrophilic, allowing it to disperse well in water and other polar solvents.

#### 3.2.3. Tunable Electronic Properties

The electronic properties of graphene oxide can be tuned by controlling the degree of oxidation and the number of oxygen-containing functional groups attached to its surface. This makes it a promising material for electronic devices and sensors.

#### 3.2.4. Mechanical Strength

Graphene oxide has excellent mechanical strength due to its strong covalent bonds between carbon atoms and the presence of oxygen-containing functional groups, which can interact with other molecules through hydrogen bonding [19,20,21].

#### 3.2.5. Applications of Graphene Materials in Vaccine Design

Graphene and its derivatives have shown potential in vaccine design due to their unique properties, which can help improve vaccine efficacy, stability, and safety.

#### 3.2.6. Antigen Delivery

Graphene-based materials can serve as efficient antigen delivery systems, as they can load and protect antigens from degradation. The large surface area and high loading capacity of graphene enable it to carry multiple antigens, adjuvants, or other immunomodulatory molecules, facilitating targeted delivery to immune cells and enhancing immune responses [22]. The large surface area and high adsorption capacity of graphene and graphene oxide allow them to effectively bind and carry vaccine antigens, such as proteins, peptides, or even nucleic acids (DNA or RNA). The adsorption of antigens onto the graphene surface can be facilitated through various interactions, including electrostatic, hydrophobic, and π-π stacking interactions. This ability to load and transport vaccine antigens makes graphene a promising delivery platform for the targeted and efficient presentation of the antigens to the immune system [23]. Graphene-based carriers can be functionalized with specific ligands or targeting moieties to facilitate the delivery of vaccine antigens to the desired immune cells, such as antigen-presenting cells (e.g., dendritic cells, macrophages) [24]. This targeted delivery can enhance the uptake and processing of vaccine antigens by these key immune cells, leading to a more effective activation of the adaptive immune response. The unique structure and properties of graphene-based materials can mimic the size and shape of pathogens, which can help facilitate the efficient uptake and presentation of vaccine antigens by antigen-presenting cells [25]. This enhanced antigen presentation can lead to a more robust activation of T cells and B cells, ultimately resulting in a stronger and more durable immune response. As mentioned earlier, graphene and its derivatives have been explored as potential vaccine adjuvants, capable of stimulating the immune system and enhancing the immune response to the vaccine antigens. When used as a delivery platform, the adjuvant properties of graphene-based materials can further boost the effectiveness of the vaccine by activating and recruiting various immune cells. Graphene-based carriers can help protect vaccine antigens from degradation, maintaining their structural integrity and immunogenicity during storage and delivery [26,27].

#### 3.2.7. Adjuvant Properties

Graphene and its derivatives can act as adjuvants, enhancing the immune response to co-administered antigens. The immunostimulatory properties of graphene can activate immune cells, such as dendritic cells, leading to increased cytokine production and T-cell activation. Graphene and its derivatives have shown promising adjuvant properties that can be leveraged in the design and development of vaccines [28]. Graphene and graphene oxide have been observed to activate and stimulate both the innate and adaptive immune responses [29]. These materials can interact with and activate various immune cells, such as dendritic cells, macrophages, and lymphocytes, leading to the production of cytokines, chemokines, and other immune-stimulatory molecules. This immune-stimulating capability of graphene-based materials can potentially enhance the efficacy of vaccines by boosting the body’s protective immune response against the target pathogen [30]. The unique structure and size of graphene-based materials can mimic the characteristics of pathogens, facilitating the efficient uptake and presentation of vaccine antigens by antigen-presenting cells (APCs). This enhanced antigen presentation can lead to a more robust activation of T cells and B cells, ultimately resulting in a stronger and more durable immune response [31]. The adjuvant properties of graphene-based materials can be attributed to several mechanisms, including the following:

- Activating pattern recognition receptors (PRRs) on immune cells, triggering signaling cascades that promote immune responses.
- Inducing inflammasome activation, leading to the release of pro-inflammatory cytokines.
- Enhancing the recruitment and activation of immune cells at the site of vaccine administration.
- Improving the uptake and processing of vaccine antigens by APCs.

The surface of graphene can be functionalized with various molecules, such as targeting ligands or immunomodulatory agents, to further enhance the vaccine’s efficacy and specificity. This versatility allows for the development of tailored graphene-based adjuvants that can be optimized for different vaccine formulations and target pathogens. Graphene-based materials can be combined with other adjuvants or delivery systems to create synergistic effects, potentially leading to even more potent and effective vaccine formulations. The unique properties of graphene can complement and enhance the performance of other adjuvant technologies, resulting in improved immune responses and protective efficacy [26,32,33].

Based on search data in PubMed, the application of graphene materials in vaccine design is summarized in Table 1.

## 4. Controlled Release

Graphene-based nanomaterials can be engineered to enable the controlled release of vaccine components, allowing for sustained and targeted delivery to immune cells. This can help maintain vaccine efficacy over time and reduce the need for frequent booster doses. Graphene and its derivatives have been explored for their potential to enable the controlled release of vaccine components, which can be a valuable feature in vaccine design and development [44].

Graphene-based materials, such as graphene oxide and reduced graphene oxide, can effectively encapsulate and load various vaccine components, including antigens, adjuvants, and immunomodulatory agents [45]. The large surface area and versatile surface chemistry of graphene allow for the adsorption or attachment of these vaccine components, facilitating their incorporation into the graphene-based delivery system [46]. The release of the encapsulated vaccine components from the graphene-based delivery system can be controlled and tailored through various mechanisms, such as the following:

- pH-responsive release: Graphene-based materials can be designed to release the cargo in response to changes in pH, which can occur in different physiological environments or upon internalization by cells [47].
- Enzymatic or redox-triggered release: The release can be triggered by the presence of specific enzymes or changes in redox conditions, which can be exploited to target specific cellular or extracellular environments.
- Stimuli-responsive release: External stimuli, such as temperature, light, or magnetic fields, can be used to control the release of vaccine components from the graphene-based delivery system [27].

The controlled release capabilities of graphene-based systems can enable the sustained and targeted delivery of vaccine components to the desired site of action, such as immune cells or lymphoid tissues [48]. This sustained release can help maintain therapeutic levels of the vaccine components over an extended period, potentially leading to improved immune responses and prolonged protection. Targeted delivery can enhance the uptake and presentation of vaccine components by the appropriate immune cells, further optimizing the vaccine’s effectiveness. The encapsulation of vaccine components within the graphene-based delivery system can help protect them from degradation or premature release, improving the overall stability and shelf-life of the vaccine formulation. The barrier properties and structural integrity of graphene-based materials can contribute to the preservation of the vaccine components’ integrity during storage and transportation. The controlled release properties of graphene-based systems can be tailored by modifying the composition, surface functionalization, and structural features of the graphene-based materials. This versatility allows for the development of customized vaccine delivery systems that can be optimized for specific vaccine formulations, target pathogens, or desired release kinetics [39].

## 5. Improved Vaccine Stability

Graphene materials can enhance vaccine stability by protecting vaccine components from degradation, maintaining their structural integrity, and extending their shelf-life [30]. This can reduce the need for frequent replenishment and ensure a consistent supply of effective vaccines, especially in resource-limited settings [49]. Graphene and its derivatives have shown promising potential to enhance the stability of vaccine formulations, which is a crucial aspect of vaccine design and development. Graphene and graphene oxide possess excellent thermal conductivity, which can help maintain a stable temperature environment for vaccines during storage and transportation. This property can be beneficial in reducing the degradation of vaccine components, such as proteins and nucleic acids, that are sensitive to temperature changes [50]. Graphene-based materials can act as thermal regulators, helping to maintain the optimal temperature range for the vaccine formulation and preventing thermal-induced damage. Graphene and its derivatives are highly impermeable to gases and liquids, making them effective barriers against moisture and oxidation. This barrier property can help protect vaccine components from environmental factors that could lead to degradation, such as humidity, oxygen, and UV radiation. Encapsulating or coating vaccine formulations with graphene-based materials can enhance their resistance to these environmental stresses, improving the overall stability of the vaccine. The large surface area and versatile surface chemistry of graphene allow it to effectively adsorb and interact with vaccine components, such as proteins, peptides, and nucleic acids. This adsorption can help stabilize the vaccine components, preventing them from undergoing structural changes or aggregation during storage. Graphene-based materials can also act as carriers or delivery platforms for vaccine components, further enhancing their stability and maintaining their integrity. Graphene-based materials can be incorporated into vaccine formulations, either as part of the adjuvant system or as excipients, to improve the overall stability and shelf-life of the vaccine. The unique properties of graphene, such as its ability to form stable dispersions and interact with various biomolecules, can be leveraged in the development of novel vaccine formulations with enhanced stability. The stabilizing properties of graphene-based materials can be tailored and optimized for specific vaccine formulations and target pathogens. The functionalization of the graphene surface with various molecules or coatings can further enhance the stability-improving capabilities of graphene-based components [36,51,52].

## 6. Mucosal Vaccines

Graphene-based materials can be designed to target mucosal surfaces, enabling the development of mucosal vaccines that can induce local and systemic immune responses. This can provide better protection against pathogens that enter the body through mucosal surfaces, such as the respiratory or gastrointestinal tract. Mucosal vaccines, which target the body’s mucosal surfaces, such as the respiratory, gastrointestinal, and urogenital tracts, offer several advantages over traditional parenteral (injectable) vaccines [53]. Graphene-based materials, particularly graphene oxide, have been shown to possess mucoadhesive properties, allowing them to adhere to and interact with mucosal surfaces [54]. This mucoadhesive behavior can facilitate the prolonged residence time of vaccine components at mucosal sites, enhancing their absorption and interaction with the underlying immune cells. Graphene-based carriers can be used to deliver vaccine antigens to mucosal surfaces, targeting the specialized immune cells located in these areas, such as M cells and dendritic cells [55]. The unique structure and size of graphene-based materials can mimic the characteristics of pathogens, which can improve the uptake and presentation of vaccine antigens by mucosal immune cells, leading to a more robust immune response. Graphene and its derivatives have demonstrated the ability to stimulate the mucosal immune system, acting as potential adjuvants for mucosal vaccines [49]. The interaction of graphene-based materials with mucosal immune cells can trigger the production of cytokines, chemokines, and other immune-stimulatory factors, enhancing the overall immune response to vaccine antigens. Graphene-based materials can help protect vaccine components from degradation in harsh mucosal environments, which are often characterized by enzymatic activity, pH changes, and other factors that can compromise the stability of the vaccine [56]. The barrier properties and controlled release capabilities of graphene-based systems can facilitate the sustained delivery of vaccine components to the target mucosal sites, improving the vaccine’s effectiveness. The surfaces of graphene-based materials can be functionalized with various targeting ligands or immunomodulatory agents to enhance the specificity and effectiveness of mucosal vaccine formulations. This versatility allows for the development of tailored graphene-based mucosal vaccine systems that can be optimized for different routes of administration, target pathogens, or desired immune responses [57].

## 7. Needle-Free Vaccination

Graphene materials can facilitate the development of needle-free vaccination methods, such as microneedle patches or nanoparticle-based aerosols, which can improve vaccine acceptance, reduce the risk of needle-stick injuries, and simplify vaccine administration [58]. So, graphene and its derivatives have been explored as part of the development of needle-free vaccination approaches, which offer several potential advantages over traditional needle-based vaccination methods [59]. Graphene-based materials can be incorporated into microneedle array patches, which are miniaturized, painless needles that can be applied to the skin. The incorporation of graphene can enhance the mechanical strength and durability of the microneedles, allowing for efficient penetration of the skin and the controlled delivery of vaccine components [60]. Graphene’s high electrical conductivity can also enable the use of these microneedle arrays for electroporation-assisted vaccine delivery, further enhancing the uptake of vaccine antigens. The unique properties of graphene, including its high surface area and ability to interact with the skin’s barrier layers, can help improve the permeation and transport of vaccine antigens through the skin. This transdermal approach can provide a non-invasive and potentially more patient-friendly method of vaccine administration. As discussed earlier, graphene-based materials have shown promise in the development of mucosal vaccines, which can be administered through routes like the nasal, oral, or pulmonary mucosa. These needle-free mucosal vaccine delivery methods can stimulate local and systemic immune responses, potentially offering advantages over traditional injectable vaccines [61,62]. Graphene’s mucoadhesive properties and ability to interact with mucosal immune cells can contribute to the effectiveness of these needle-free mucosal vaccine approaches. Graphene-based materials can be incorporated into the vaccine formulation to improve the stability and shelf-life of the vaccine components, as discussed previously. This enhanced stability can be particularly beneficial for needle-free vaccine delivery methods, where the vaccine may be exposed to various environmental factors during storage and transportation. Needle-free vaccination methods, such as those utilizing graphene-based technologies, can potentially provide a more comfortable and less intimidating experience for vaccine recipients, especially children and individuals with needle phobia. Improved patient acceptance and compliance with vaccination schedules can lead to higher vaccination rates and better public health outcomes. While the research on graphene-based needle-free vaccination approaches is still in the early stages, the unique properties of graphene offer promising opportunities to develop innovative and user-friendly vaccine delivery methods. Continued advancements in this field, along with rigorous safety and efficacy evaluations, will be crucial for translating these graphene-based needle-free vaccine technologies into practical and widely accessible solutions [29,63,64].

## 8. Disadvantages of Graphene in Vaccine Design

While graphene and its derivatives have shown promising applications in vaccine design, there are also some potential disadvantages and challenges associated with the use of graphene in this context.

**Biocompatibility and toxicity concerns:** The biocompatibility and potential toxicity of graphene-based materials are still not fully understood, particularly when used in direct contact with biological systems and the human body. Factors such as the size, shape, surface chemistry, and purity of graphene can influence its interactions with cells and tissues, which may lead to adverse effects. Extensive biocompatibility testing and safety evaluations are necessary to ensure the safe use of graphene-based components in vaccine formulations. Graphene-based materials can have potentially toxic effects on cells and tissues, depending on their size, shape, surface chemistry, and dose. For example, some studies have reported that graphene can induce oxidative stress, inflammation, and genotoxicity in cells, which may raise safety concerns for their use in vaccines.

The biocompatibility and biodegradability of graphene-based materials are crucial factors that determine their safety and efficacy in vivo. However, the long-term fate and potential accumulation of graphene-based materials in the body remain unclear, which may raise concerns about their potential adverse effects on human health [30,65].

**Scalability and manufacturing challenges:** The large-scale production of high-quality and consistent graphene-based materials for vaccine applications can be challenging and may require complex and costly manufacturing processes. Ensuring the reproducibility and quality control of graphene-based vaccine components during mass production can be a significant hurdle in translating these technologies into commercial products. The large-scale production and standardization of graphene-based materials with consistent quality, purity, and properties can be challenging, which may hinder their translation into clinical applications. Moreover, the functionalization and modification of graphene-based materials can further complicate their production and standardization processes [28,66].

**Regulatory hurdles:** The incorporation of novel materials like graphene into vaccine formulations may face additional regulatory scrutiny and approval processes, which can slow down the development and commercialization of graphene-based vaccine products. Regulatory agencies may require extensive safety and efficacy data to demonstrate the suitability and reliability of graphene-based vaccine components [28].

**Limited clinical evidence:** The clinical data on the use of graphene-based materials in vaccine applications are still limited, as most of the research has been conducted at the preclinical or early clinical stages. The lack of robust clinical trials and long-term follow-up data may hinder the widespread adoption and acceptance of graphene-based vaccine technologies by healthcare providers and the public [39].

**Potential immunogenicity:** Graphene-based materials, if not properly designed and formulated, may have the potential to elicit unintended immune responses or trigger adverse reactions in some individuals. Careful evaluation of the immunogenicity of graphene-based vaccine components is necessary to ensure their safety and avoid any potential immune-related complications. Graphene-based materials may have potential immune-suppressive effects, depending on their properties and doses. For example, some studies have reported that graphene can suppress the activation and proliferation of immune cells, such as T cells and B cells, which may compromise the efficacy of vaccines [48,67].

**Cost and accessibility:** The production and implementation of graphene-based vaccine technologies may initially be more expensive compared to traditional vaccine formulations, which could limit their accessibility, especially in resource-limited settings. Efforts to scale up production and optimize the manufacturing processes will be crucial for making graphene-based vaccines more cost-effective and widely available [68,69].

**Limited understanding of structure–activity relationships**: The structure–activity relationships of graphene-based materials, such as the effects of their size, shape, surface chemistry, and functionalization on their immunomodulatory properties, are not yet fully understood. This may limit the rational design and optimization of graphene-based materials for specific vaccine applications [70,71].

**Figure 2:**
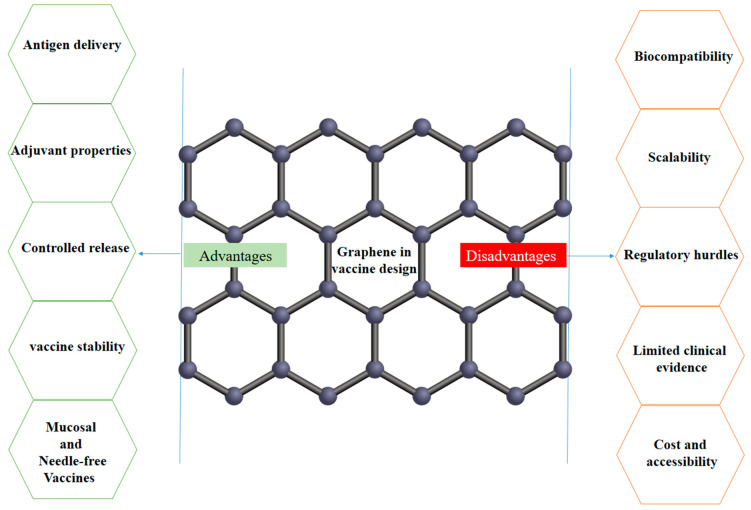
The advantages and disadvantages of graphene in vaccine design.

Figure 2 shows the advantages and disadvantages of graphene in vaccine design.

## 9. Future Trends in the Area of Graphene

Future trends in the area of graphene materials in vaccine design and development include the following:

1. Personalized vaccines: Graphene-based materials can be tailored to deliver personalized vaccine formulations based on an individual’s genetic makeup, immune response, and disease susceptibility. This can lead to more effective and targeted vaccination strategies.
2. Multivalent vaccines: Graphene’s ability to carry multiple antigens or adjuvants simultaneously can enable the development of multivalent vaccines that protect against multiple pathogens or strains, reducing the need for multiple vaccine doses.
3. Nanoparticle-based vaccines: Graphene-based nanoparticles can be engineered to deliver vaccine components directly to immune cells, enhancing antigen uptake and presentation. This can lead to more potent and durable immune responses.
4. Smart vaccine systems: Graphene-based materials can be integrated with sensors or other smart technologies to monitor vaccine efficacy, storage conditions, and immune responses in real time. This can help optimize vaccine formulations and administration strategies.
5. Next-generation adjuvants: Graphene-based materials can be functionalized with novel immunomodulatory agents or combined with other adjuvants to enhance the immune response to vaccines. This can lead to the development of more potent and effective adjuvant systems.
6. Needle-free vaccine delivery: Graphene-based materials can be used to develop innovative needle-free vaccination methods, such as aerosolized or oral vaccine formulations. This can improve vaccine acceptance and simplify vaccine administration, particularly in resource-limited settings.
7. Rapid vaccine development: Graphene’s ability to rapidly adsorb and deliver vaccine components can accelerate vaccine development and production, enabling faster responses to emerging infectious diseases or pandemics.
8. Vaccine thermostability: Graphene-based materials can improve the thermostability of vaccines, reducing the need for cold-chain storage and increasing vaccine accessibility in regions with limited infrastructure.
9. Immunomodulatory therapies: Graphene-based materials can be used to develop novel immunomodulatory therapies that target specific immune pathways or cells, providing new treatment options for autoimmune diseases or cancer.
10. Combination therapies: Graphene-based materials can be combined with other nanomaterials or biomolecules to create hybrid vaccine systems with enhanced properties, such as improved antigen delivery, adjuvant activity, or controlled release capabilities.

Therefore, the future of graphene materials in vaccine design and development holds great promise, with the potential to revolutionize vaccination strategies and improve global health outcomes.

## 10. Conclusions

In conclusion, graphene and its derivatives have shown significant potential in vaccine design, offering advantages in antigen delivery, adjuvant properties, controlled release, improved vaccine stability, mucosal vaccines, and needle-free vaccination methods. The unique properties of graphene, such as its high surface area, hydrophilicity, tunable electronic properties, and mechanical strength, make it an attractive material for vaccine development.

Graphene-based materials can serve as efficient antigen delivery systems, effectively loading and protecting antigens from degradation and facilitating targeted delivery to immune cells. The adjuvant properties of graphene can stimulate both innate and adaptive immune responses, enhancing the efficacy of vaccines.

Graphene-based systems can enable the controlled release of vaccine components, maintaining therapeutic levels over time and reducing the need for frequent booster doses. They can also enhance vaccine stability by protecting vaccine components from degradation and maintaining their structural integrity.

Graphene-based materials can be designed to target mucosal surfaces, enabling the development of mucosal vaccines that can induce local and systemic immune responses. They can also facilitate the development of needle-free vaccination methods, such as microneedle patches or nanoparticle-based aerosols, which can improve vaccine acceptance and simplify vaccine administration.

Overall, graphene and its derivatives have the potential to revolutionize vaccine design and development, offering innovative solutions to address the challenges associated with traditional vaccine technologies. Continued research and development in this field will be crucial for unlocking the full potential of graphene in vaccine design and optimization, ultimately contributing to the success of vaccination programs worldwide.

## Figures and Tables

**Table 1:** Application of graphene materials in vaccine design.

Author	Title	Results	Ref.
Alphandéry	Nano dimensions/adjuvants in COVID-19 vaccines	The proposed COVID-19 vaccines contain various vaccine active principles and adjuvants such as graphene oxide that can potentially enhance their effectiveness and reduce the necessary dose, thereby facilitating mass vaccination and potentially bringing an end to the COVID-19 crisis. These adjuvants, inspired by cancer nano-vaccines, may improve the vaccine’s benefit-to-risk ratio and modulate the immune response by targeting specific regions and adjusting physical properties.	[34]
Huang et al.	A self-assembled graphene oxide adjuvant induces both enhanced humoral and cellular immune responses in influenza vaccine	Self-assembled nanoparticles based on graphene oxide quantum dots with the adjuvant ZnGC-R were designed for influenza vaccines, enhancing antigen utilization, DC recruitment, and antigen-presenting cell activation. This novel adjuvant induces robust CD4+ and CD8+ T-cell responses, promotes CD26+ B-cell proliferation, elicits higher levels of hemagglutination-inhibiting antibodies, and significantly boosts IgG responses, leading to 100% in vivo protection against H1N1 influenza.	[26]
Yan et al.	Chitosan-Functionalized Graphene Oxide as a Potential Immunoadjuvant	The study introduced CS-functionalized GO (GO-CS) as a safe and effective nanoadjuvant for vaccines, showing improved biocompatibility and immune response activation compared to non-functionalized GO. The GO-CS adjuvant also exhibited a smaller size, positive charge, and enhanced thermal stability, making it a promising candidate for vaccine development and immunotherapy.	[35]
Wang et al.	Alum-functionalized graphene oxide nanocomplexes for effective anticancer vaccination	The study describes the development of an aluminum-based adjuvant in the form of AlO(OH)-modified graphene oxide (GO) nanosheets (GO-AlO(OH)) as an effective vaccine adjuvant. GO-AlO(OH) not only maintains the induction of humoral immunity but also elicits a cellular immune response. Antigen-loaded GO-AlO(OH) nanocomplexes enhance the cellular uptake and cytosolic release of antigens, promote dendritic cell maturation, stimulate higher antigen-specific IgG titers and robust CD4+ and CD8+ T lymphocyte responses, and inhibit tumor growth in vivo. This novel formulation may serve as a facile and efficient approach for effective anticancer vaccination.	[36]
Gao et al.	Developing an efficient MGCR microneedle nanovaccine patch for eliciting Th 1 cellular response against the SARS-CoV-2 infection	The study aimed to enhance the immunogenicity of subunit vaccines for COVID-19 and other epidemics by creating a CpG 1018- and graphene oxide-based bi-adjuvant system. This system successfully delivered the Receptor-Binding Domain (RBD) of the SARS-CoV-2 spike protein, resulting in the graphene oxide-based complex adjuvant nanovaccine (GCR). The GCR nanovaccine induced robust antibody responses and Type 1 Cellular responses in CD8+ T cells. Furthermore, a microneedle patch vaccine (MGCR) was developed based on the GCR vaccine, which produced a similar antibody response, maintained high antibody levels over time, and increased the Tcm proportion in the mouse spleen. The MGCR vaccine also exhibited improved storage stability and could be administered without medical staff, enhancing vaccine distribution efficiency. This innovative vaccine system offers a promising approach to combating SARS-CoV-2 infection and future pandemics.	[37]
Zhao et al.	Novel use of graphene oxide quantum dots in a pickering emulsion as a Chlamydia trachomatis vaccine adjuvant	The study investigated the use of graphene oxide quantum dots (GOQDs) as a surfactant substitute in Pickering emulsions for the Chlamydia trachomatis Pgp3 recombinant vaccine. GOQD-stabilized Pickering emulsion (GQPE) enhances immune responses, prolonging the immune response and promoting dendritic cell recruitment. It is found to be a safe and effective adjuvant, stimulating various immune responses and improving immune response duration.	[38]
Xu et al.	Functionalized graphene oxide serves as a novel vaccine nano-adjuvant for robust stimulation of cellular immunity	The study explored graphene oxide (GO) as a vaccine adjuvant for immunotherapy using urease B (Ure B) as the model antigen. Dual-polymer-modified GO (GO-PEG-PEI) was developed as a positive modulator, promoting dendritic cell maturation and cytokine secretion. GO-PEG-PEI also effectively transports antigens into dendritic cells, making it a promising vaccine adjuvant. Compared to free Ure B and an aluminum-adjuvant-based vaccine (Alum-Ure B), GO-PEG-PEI-Ure B induces stronger cellular immunity, showcasing its potential in cancer immunotherapy. The study highlights the critical role of surface chemistry in the rational design of nanoadjuvants.	[39]
Jiao et al.	Lentinan-functionalized graphene oxide hydrogel as a sustained antigen delivery system for vaccines	The study introduced a lentinan-functionalized graphene oxide hydrogel (LNT-GO Gel) as a sustained antigen delivery system for vaccines. The LNT-GO Gel effectively encapsulates and releases antigens, enhances immune responses, and displays favorable safety and biodegradability, highlighting its potential as an adjuvant delivery platform for subunit vaccines.	[40]
Liu et al.	Lentinan-Functionalized Graphene Oxide Is an Effective Antigen Delivery System That Modulates Innate Immunity and Improves Adaptive Immunity	The study synthesized graphene oxide grafted with lentinan (GO-LNT) to enhance antigen uptake in macrophages, resulting in sustained long-term immune responses and increased IgG levels compared to GO/OVA. GO-LNT demonstrates potential as a safe and effective vaccine delivery system and an excellent adjuvant for eliciting long-term immune memory and boosting both cellular and humoral immunity.	[41]
Yang et al.	Poria cocos polysaccharide-functionalized graphene oxide nanosheet induces efficient cancer immunotherapy in mice	The abstract describes the creation of nanocomplexes named nsGO/PCP/OVA for cancer immunotherapy, using graphene oxide nanosheets as carriers for ovalbumin (OVA) and Poria cocos polysaccharides (PCPs). These nanocomplexes were successful in activating specific immune responses, inducing dendritic cell maturation, and effectively combating tumor growth, both preventatively and therapeutically. Overall, this nanovaccine platform shows great potential for enhancing anti-tumor immunity and improving cancer immunotherapy.	[42]
Zhao	Preparation of graphene oxide-stabilized Pickering emulsion adjuvant for Pgp3 recombinant vaccine and enhanced immunoprotection against Chlamydia Trachomatis infection	Researchers developed a graphene oxide-stabilized Pickering emulsion (GPE) to serve as a novel adjuvant for the Chlamydia trachomatis Pgp3 vaccine, demonstrating superior immune responses, including enhanced macrophage polarization and recruitment, increased cytokine production, elevated levels of specific immunoglobulins, and improved protection against Chlamydia muridarum infection compared to the vaccine with traditional adjuvants.	[43]
